# Who Should Access Closed-Loop Technology? A Qualitative Study of Clinician Attitudes in England

**DOI:** 10.1089/dia.2019.0380

**Published:** 2020-04-27

**Authors:** Conor Farrington, Roman Hovorka, Helen R. Murphy

**Affiliations:** ^1^ THIS Institute (The Healthcare Improvement Studies Institute), University of Cambridge, Cambridge, United Kingdom.; ^2^ Wellcome Trust-Medical Research Council Institute of Metabolic Science, University of Cambridge, Cambridge, United Kingdom.; ^3^ Norwich Medical School, University of East Anglia, Norwich, United Kingdom.; ^4^ Cambridge University Hospitals NHS Foundation Trust, Cambridge, United Kingdom.; ^5^ Department of Women and Children's Health, King's College London, London, United Kingdom.

**Keywords:** Insulin pumps, Continuous glucose monitoring, Closed-loop systems, Type 1 diabetes

## Abstract

***Background:*** Clinicians mediate access to closed-loop technology for people with diabetes. Consequently, their attitudes regarding appropriate levels of closed-loop usage will play a key role in future adoption processes. This study aimed to explore clinician attitudes toward future mainstream closed-loop usage in England.

***Materials and Methods:*** We conducted 36 semistructured interviews with clinicians from a range of professional backgrounds working in outpatient clinics in England. Interview topics included clinicians' views on future pathways for closed-loop use and attitudes toward the predictability of users' technology experiences, a key factor in eligibility decision making. We analyzed transcripts using thematic and framework approaches.

***Results:*** Clinicians exhibited a range of opinions regarding future eligibility for closed-loop technology. We identified three key strands of clinician opinion, envisaging (1) tighter access for closed loop (*n* = 10), citing funding challenges and issues arising from user overconfidence or negative technology attitudes; (2) similar access to closed loop as for current diabetes technologies (*n* = 15), on the grounds that future funding and access pathways will be similar to current arrangements; and (3) wider access for closed-loop technologies (*n* = 9), given the potential for significant and widespread benefits arising from closed-loop usage, including downstream cost savings alongside improved glycemic control.

***Conclusions:*** Clinicians expressed a range of opinions encompassing continuity with current diabetes technologies, while others envisaged either tighter or more liberal access for closed-loop systems. To optimize technology adoption and equitable uptake, future implementation pathways should consider clinician attitudes toward technology use and access.

## Introduction

Closed-loop (or “artificial pancreas”) systems link wearable continuous glucose monitor (CGM) sensors to algorithms that process CGM data and issue frequently updated infusion commands to single-hormone (insulin) or dual-hormone (insulin and glucagon) pumps.^1^ Multiple closed-loop systems have been developed, and their safety and efficacy have been evaluated in numerous studies, generating impressive results in terms of glycemic control.^2,3^ Commercially available systems such as the FDA-approved Medtronic 670G, a hybrid system requiring user input for tasks such as prandial bolusing, raise the possibility of widespread access to closed-loop technology.^4^ In resource-constrained settings, however, financial pressures may generate incentives for limiting access,^5^ particularly if the greater complexity of closed-loop systems requires additional health service expenditure on training, staffing, and logistical support. In addition, recent data showing high levels of attrition in 670G usage in the USA highlight the user-centered challenges that may arise with widespread hybrid closed-loop usage.^6^ While highly specific to 670G systems, these data nevertheless echo previous psychosocial research reporting burdens as well as benefits for closed-loop users.^7^ While 670G and (increasingly) home-made closed-loop systems are used by growing numbers of users, national and international eligibility guidelines have yet to be formulated.^8^ Consequently, while the automated nature of closed-loop technology promises improved control for people with type 1 diabetes, debate continues regarding appropriate levels of closed-loop usage in mainstream care.

Clinician attitudes are currently a neglected aspect of this debate, yet evidence from implementation research shows that technology adoption is more successful when a range of stakeholder perspectives are taken into account.^9^ A recent review, for example, emphasizes the potential for clinicians to both enable and hinder technology adoption, stating that, “[a]cceptance [of new technologies] by professional staff may be the single most important determinant of whether a new technology-supported service succeeds or fails at a local level.”^9^

This importance arises by clinicians' privileged decision-making positions, from which they adjudicate whether individuals are legitimate candidates for specific treatments or technologies.^10^ In the specific context of diabetes technology, researchers have begun to explore how clinicians mediate access to currently available devices such as pumps, CGM, and sensor-augmented pumps (including low glucose suspend systems), for example, by supplementing frequently ambiguous formal criteria in national guidelines with informal criteria based on clinical experience.^11^ A study of diabetes specialist nurses and dieticians involved in the REPOSE trial found that clinicians had strong, yet often inaccurate beliefs regarding the suitability of individuals for pump use.^12^ While these predictions about the likely character of users' future technology experiences were overridden in REPOSE by randomization to pump or multiple daily injection, participating clinicians acknowledged that their views about individuals' suitability for specific devices did influence decision making about technology eligibility outside trial conditions.

While previous researchers have explored clinician attitudes to other aspects of technology in type 1 diabetes care, little is known on clinician views about access to closed-loop technology.^13–17^ To address this gap, we undertook a qualitative study of clinician attitudes in England, guided by the following research question: what are clinicians' attitudes toward user eligibility for future closed-loop systems in mainstream care in England?

## Research Design and Methods

### Design

Qualitative study using semistructured interviews.

### Setting

We carried out 36 interviews with clinicians working at five hospital diabetes outpatient clinics serving adult (two clinics), pregnant (two clinics), and pediatric (one clinic) populations with type 1 diabetes in three hospitals in England, chosen to provide a range of geographical and socioeconomic contexts. Hospital 1 (pregnancy and pediatric clinics) is a large teaching hospital in an affluent area in the East of England; Hospital 2 (pregnancy and adult clinics) is a teaching hospital in a less affluent area, situated in East Anglia; and Hospital 3 (adult clinic) is a large teaching hospital in the north of England. A sixth clinic serving pediatric patients in Hospital 3 declined to participate in the study. All three hospitals had been involved in diabetes technology trials.

### Participant recruitment

Following ethics approval, we received permission from local NHS Trusts to approach members of outpatient clinic staff for interview. We contacted potential participants by email, with a participant information sheet and consent form, and offered all participants an interview in person or by telephone at a convenient time, date, and place. In addition, we used a snowball sampling approach to identify additional staff for interview, asking participants for recommendations of other suitable candidates. We attempted to interview clinicians from a range of professional backgrounds (see Table 1 for participant characteristics).

**Table 1.** Participant Characteristics

Professional background		General medicine/endocrinology (PHYS)	Nursing (NU)	Dietetics (DI)	Obstetrics (OBS)	Midwifery (MW)	Anesthesiology (ANAEST)	Psychology (PSYCH)	Total
Location	Outpatient clinic population								
Hospital 1	Pregnancy (PR)	1	2	2	2	1	/	/	8
	Pediatric (PA)	6	2	3	/	/	/	1	12
Hospital 2	Pregnancy (PR)	2	3	1	1	1	1	/	9
	Adult (AD)	4	/	/	/	/	/	/	4
Hospital 3	Adult (AD)	2	1	/	/	/	/	/	3
Total		15	8	6	3	2	1	1	36

Participants are identified using the following naming convention: hospital number/clinic population/profession/number of interviewees within clinic. Abbreviations for clinic population and profession are given in parenthesis in relevant headings of Table 1.

### Data collection

[Name removed for peer review] conducted interviews in person (*n* = 29) and by telephone (*n* = 7), between October 2017 and June 2018. All participants gave informed consent to participate and to allow digital recording and verbatim transcription of interviews by a trusted agency. We used a semistructured topic guide informed by relevant literature and designed to allow for the exploration of a range of issues relevant to diabetes technology. In the context of guidelines, our topic guide focused on technology access pathways and on clinician attitudes to the predictability of technology use by people with diabetes (see Supplementary Data for detailed topic guide). Interviews lasted between 28 and 73 min, with an average time of 47.5 min.

### Data analysis

We analyzed the data using a combination of thematic and framework analysis approaches. Initial coding of interview transcripts took place alongside data collection to identify key themes and generate a provisional coding structure. We then utilized this provisional structure to undertake an initial thematic analysis, using QSR NVivo (Version 12) software (see Supplementary Table S1 for details of coding structure). Our thematic analysis approach used a six-stage inductive approach: familiarization with the data; generating initial codes; searching for themes; reviewing themes; defining and naming themes; and producing an overall analysis.^17^

We then supplemented our thematic analysis with framework analysis, a more deductive method involving the use of a matrix with cells into which summary qualitative data are entered by category (rows) and cases (columns).^18^ This allowed us to identify and explore patterns (categories) that cut across individual clinician attitudes (cases). For this study, we focused on two key areas: clinician expectations regarding future access pathways to closed loop and clinician perspectives on the predictability of technology use by people with diabetes, including closed loop alongside older technologies such as pumps and CGM. C.F. conducted the analyses, later discussing them with H.R.M. and R.H. to establish reader agreement.

## Results

Our findings are presented in three main sections, relating, respectively, to clinician viewpoints suggesting that: (1) access to closed loop will be tighter than eligibility for currently available technologies (*n* = 10); (2) eligibility for closed loop will be similar to eligibility for currently available CGM and CSII technologies (*n* = 14); and (3) eligibility will be wider than eligibility for currently available technologies (*n* = 9). Clinicians invoked a range of reasons to support each view, as indicated by subheadings in each section.

In terms of professions, most physicians expected looser access arrangements and most nurses mostly expected looser or similar access arrangements; otherwise, no clear patterns emerged across different professions. Similarly, no clear patterns emerged across different hospitals or clinics serving different populations.

### Tighter eligibility constraints for closed loop

Several interviewees (*n* = 10) recommended limiting eligibility to users who will clearly benefit from closed-loop technology, raising the possibility of stricter constraints than those currently in place for pumps and CGM. Clinicians varied in the kinds of reasons they gave for expressing this point of view, including user-centered technical challenges, psychological impacts, unrealistic expectations, and funding issues.

#### Technical challenges

Some clinicians framed tighter eligibility constraints in terms of the greater technical challenges and user input required for successful operation of closed-loop systems, thus reserving access for highly motivated protechnology users. One physician, for example, described closed-loop technology as a “step higher” and therefore requiring “more work” than existing devices (1/PA/PHYS/2), while a nurse in the same pediatric clinic suggested that “some families may struggle with the whole technology side of things… [A] lot of families [of children with diabetes] don't get on with technology” (1/PA/PHYS/5). In this context, clinicians expressed concerns that operation of closed-loop systems without, for example, careful manual premeal bolusing could lead to suboptimal outcomes for users. One physician described this scenario with a motoring analogy: “we're giving you [the user] a Ferrari, you're choosing not to get out of second gear” (2/AD/PHYS/1).

#### Psychological impacts

Other clinicians worried about the potential impacts of closed-loop technology on some users' psychological status, describing this as “the biggest unknown” and asking if these new systems might make users “more anxious” (3/AD/PHYS/1) as a result of surrendering control. As one physician put it, “[T]here are lots of people that will … say that they prefer to have control, i.e., do their own injections. They don't want to give that control over to an inanimate object” (2/PR/PHYS/2).

#### Unrealistic expectations

Interviewees also expressed psychological concerns regarding protechnology users, suggesting that some users might place undue confidence in closed-loop systems and/or engage less proactively in self-care. One physician described this concern in terms of those who might “plug [the closed-loop system] in and forget, [thinking] you don't have to do anything else, [whereas] you probably need to do a lot more than you would normally be doing” (2/AD/PHYS/2). Similarly, a psychologist working in a pediatric clinic highlighted the danger of unrealistic expectations: “[some families] just think this device is just going to do everything and actually that's not the case” (1/PA/PSYCH/12). In such contexts, clinicians expressed concern that some protechnology users may not notice technical glitches, with potentially negative impacts on their health: “you need to question it, you need to recognize that it might not be calibrated… that's the worry [with] the [users] who just go, ‘skippety skip, off we go’” (1/PA/PHYS/6).

#### Funding challenges

A number of clinicians raised issues regarding funding, which they saw as likely to place tight limits on future closed-loop access. Specifically, some feared greater restrictions on closed-loop usage than pumps because of the higher cost of newer systems: “I'm pretty sure it'll only be given to a very small percentage of [patients]” (1/PR/DI/11). Similarly, one nurse stated that “we need more, other criteria to define who needs to be [using closed loop]… because mainly I think it will be an expensive system… And it's more complicated probably than the pump or the [CGM] sensor per se” (1/PA/NU/2). One physician expressed the view that future National Institute for Health and Care Excellence (NICE) guidelines regarding closed-loop access are likely to be stringent:

You're talking about what's going to be an expensive gadget…I guess in future if it comes in the UK … it won't be very lenient criteria [but rather] very stringent criteria, for example life-threatening hypoglycaemia.

2/AD/PHYS/1

### Similar eligibility constraints for closed loop

While some clinicians advanced the view that future access to closed loop may need to be limited from both user welfare and funding perspectives, a substantial number (*n* = 15) thought that levels and means of access would reflect current access arrangements for pumps and CGM. Clinicians in this group advanced a range of reasons in support of this perspective, including organizational continuity, funding challenges, user engagement, and challenges surrounding predictions of user technology experience.

#### Organizational continuity

Clinicians in this group emphasized that future users will continue to gain access to diabetes technology through health service pathways similar to those currently used for technologies such as pumps and CGM, featuring “multidisciplinary team discussions” involving clinicians from a range of backgrounds and guided by clinic organizational structures, NICE eligibility criteria, and local funding arrangements (1/PA/DI/10). One dietician stated that future access “will probably be quite similar to how we [discuss] pumps. Obviously it won't be suitable for everyone, there will definitely need to be some criteria we would use” (1/PR/DI/5). In the absence of published guidelines, clinicians expressed uncertainty regarding the precise content of future eligibility criteria; one dietician suggested for instance that “I'm guessing there'll be criteria that need to be met… I don't really know yet, so I guess we'll just have to wait and see” (1/PA/DI/10).

Clinicians also envisaged similar accessibility decision making within the context of these organizational continuities. One physician, for example, envisaged prioritization of selected users for closed-loop usage in future, as currently occurs when initiating pump therapy: “Anything new, we do tend to select patients at the start, so when we started pumps, we didn't start with our high-risk patients, we started with our incredibly reliable patients” (1/PR/PHYS/1).

#### Funding challenges

A number of clinicians thought closed-loop access would be limited by funding in a similar way to current use of pumps and CGM. As one nurse put it: “I think it would be quite similar to now, in the respect that … there is always going to be financial restraints, [so] we are going to have to have these discussions… [about] who is eligible for them” (2/PR/NU/6).

#### User engagement

Clinicians in this group also emphasized continuities between access to closed loop and access to previous technologies in terms of the importance of user engagement and motivation as factors in gaining access to diabetes technology. Some clinicians suggested that users' engagement in self-care and technology use would function as a criterion for closed-loop access, as it currently does for pumps and CGM:

The people who might struggle with it… will just put the system on and ignore it and … they won't test, they won't calibrate it… [B]ut if they're not calibrated, they won't be given CGM, [and] they can't have the closed-loop system [either]. So I think it might police itself, really.

3/AD/NU/2

#### Challenges in predicting user technology experience

Clinicians drew on past experience with pump and CGM technologies to emphasize the difficulty of predicting whether individual users would react positively or otherwise to closed-loop use, which is a key constraining factor in current access decisions. One physician remarked, for example, that “I think you've got a sense who's going to do well or not, but sometimes patients prove you wrong… maybe ten per cent [or] twenty per cent [of the time]” (2/AD/PHYS/4). Similarly, a midwife in a different clinic stated that “there's that 20 per cent that, for whatever reason, whether it's us or them or their diabetes… it's just not going to work for them” (1/PR/MW/8).

Relatedly, some clinicians also highlighted the ever-present possibility of being surprised by users' technology experience, further emphasizing the constraints involved in making access decisions based on predictions of technology experience. One physician expressed surprise at the positive experiences of disadvantaged people and, by comparison, the negative experiences of more advantaged people (such as clinicians themselves): “I'm sometimes surprised at the healthcare professionals [with diabetes] who don't seem to take it very seriously [and who are] very casual and relaxed in their self-management” (2/PR/PHYS/4). Another clinician supported this view, stating: “there's always surprises in terms of user technology outcomes” (1/PA/PHYS/6), while another stressed that users' age was not, as often assumed, an infallible guide to success: “I think maybe the assumption is if somebody's younger… that they'd be okay with [technology] but sometimes it's the reverse, so you can never quite tell” (2/PR/PHYS/2). In addition, others highlighted the possibility of technology itself triggering changes in levels of user engagement. Interviewees saw this as a complicating factor in attempts to predict further use: as one nurse put it, “until we start using [closed-loop technology] I wouldn't really know who would be good with it and who wouldn't” (3/AD/NU/2).

### Looser eligibility constraints for closed loop

A number of clinicians (*n* = 9) hoped for wider usage for closed-loop systems than for currently available technologies. They based this perspective on a range of considerations, including expectations of potentially widespread benefits from closed-loop usage, the likelihood of liberal access guidelines, the potential for cost savings arising from closed-loop usage, and (in opposition to clinicians cited above) the unpredictability of user technology experience.

#### Widespread benefits

One physician described a vision of closed-loop systems as potentially benefitting up to 80% of people with type 1 diabetes:

I think there'll always be a group of patients, like 20/30 per cent, who'll be able to get better control by themselves than they would do with closed loop, but what I see closed loop doing is getting a larger range…a broader range of people to an acceptable level.

2/PR/PHYS/4

Another physician in the same clinic described closed-loop systems as “particularly good for patients who aren't very motivated … because the difficult stuff will be done for them” (2/PR/PHYS/1). Others clinicians envisaged a less universal, but still generous set of parameters; one physician stated, for instance, that “anyone whose… control is poor in spite of clinical optimization, and anyone who's got a fear [of] or… significant problems with hypoglycemia should be eligible for closed loop” (2/PR/PHYS/1).

#### Liberal access guidelines

In this light, a number of clinicians emphasized the potential for liberal eligibility guidelines for closed-loop systems. One midwife described the basis of a liberal access policy on pragmatic and evidential grounds: “[I]f we've got the reassurance that it works, then why would you be selective?” (1/PR/MW/8). Another clinician advocated universal usage if funding and wearability constraints could be overcome:

[closed-loop systems] will be good for anyone with type 1 diabetes… if money was not an issue then I think anyone would be good for it. I can't for the moment think of someone who might not want it, apart from people who might not like to be attached to something all the time.

2/PR/DI/3

Some interviewees framed a liberal approach to access in terms of ethical responsibility, for example:

[O]nce closed-loop technology really takes off, we won't have the same kind of discussions [as with pumps and CGM]… [I]f that's the therapy that's proven to the best for everyone, then it would be wrong to deny that to people.

1/PA/NU/7

#### Cost savings

In opposition to the fear (mentioned above) that high costs could limit access, some clinicians raised the possibility that improved glycemic control offered by closed-loop systems could help patients to avoid complications, which in turn would reduce health care spending. One midwife stated that “when you think of the complications, it would be a lot cheaper to [use closed-loop systems than not]” (2/PR/MW/7). From this perspective, interviewees saw high short-term costs as offset by long-term savings, again supporting a generous access and eligibility policy.

#### Beyond predictability

While some clinicians (as discussed above) saw the unpredictability of future technology outcomes as a reason to be cautious regarding user eligibility, others were inclined to be more adventurous in terms of future access, precisely because of the difficulty of predicting future technology use. One physician in a pregnancy clinic described this approach as follows: “I think it is always very hard to tell who… will do well and who won't. So… my own preference is to let people have a go and then see how they go” (2/PR/PHYS/4). Others seemed to suggest that predictability would be less important in the closed-loop context because of the potentially transformative impacts of closed-loop usage on users themselves: “any concerns that people have will disappear very quickly, once they get the faith in the technology” (3/AD/PHYS/3). Another clinician in a pregnancy clinic stated a preference for wide use regardless of predictability, since “closed loop can really help achieve [better control]” and therefore “we will want all of our women on it if it is available” (1/PR/DI/2).

## Conclusions

Our interviewees expressed a wide range of opinions regarding the question of who should access closed-loop technology. A group of 15 clinicians emphasized continuities between closed loop and preceding technologies such as pumps and CGMs. Specifically, these interviewees highlighted the continuing need for users to gain access to future technology through similar health service pathways as are currently in place, in addition to the ongoing need to take account of funding limitations, allow for unpredictable technology experiences, and prioritize access for more highly motivated users.

A larger group of 19 clinicians thought that future access arrangements would differ for closed-loop technology, but were divided as to whether access should be tighter (*n* = 10) or more liberal (*n* = 9) compared with pumps and CGM. Those who expected tighter restrictions mentioned a range of considerations, such that advanced closed-loop technology required motivated and protechnology users, but also that protechnology users who placed excessive trust in closed loop could suffer suboptimal outcomes as a result. These clinicians also suggested that higher costs associated with closed-loop systems could lead to stringent eligibility guidelines in England. Those who envisaged more liberal access, by contrast, thought that long-term benefits of widespread closed-loop usage (e.g., reduced complications) would outweigh initial investment in closed-loop provision. These interviewees saw widespread usage as justified by factors, including the clinical benefits of closed-loop usage, ethical duties to provide the best possible care, and the challenges of predicting technology use. If up to 80% of the type 1 diabetes population would benefit from closed-loop usage, as one physician suggested, it becomes less vital to predict technology outcomes for an individual.

While clinicians generally aligned themselves with perspectives envisaging tighter, similar, or looser access to future closed-loop systems, they varied in terms of the kinds of reasons they gave in support of these views. In each group, interviewees variously mentioned technical, psychological, financial, and (in the “similar” access group) organizational factors. In addition, clinicians who discussed certain factors—for example, psychological factors in the “tighter” group—did not always emphasize the same specific factors. Consequently, our interviewees demonstrated considerable diversity of opinion within, as well as between, the three main groups identified in our analysis.

Our study advances beyond previous work primarily in its attempt to elicit and explore clinician rather than user perspectives on closed-loop technology, and in its identification of the wide range of clinician opinion regarding future eligibility for closed-loop usage. Some of these attitudes align with previous research on user experience of closed-loop technology, and, in particular, with studies emphasizing a mix of potential benefits and burdens for users of closed-loop systems.^7^ Yet our interviewees also provided informative clinician perspectives on a number of further topics, including issues of short- and long-term funding, predictability of user technology experience, ethical duties of care, and the need to establish clear expectations from the outset.^19^ Most importantly, our findings demonstrate that clinicians hold widely differing views regarding future eligibility for closed-loop technology, encompassing three broad groups with tighter, similar, and looser access constraints, respectively. The range of opinions expressed suggests that closed loop has yet to attain a clear and settled “technology identity,” or a widely shared understanding of closed-loop technology.^20^ In the absence of such an identity for closed-loop systems, there is a risk of clinicians drawing on informal criteria to limit or expand access, as with current insulin pumps and CGM technologies.^11–12^ Specifically, our findings regarding prioritized technology access for highly motivated users raise concerns regarding clinician gatekeeping in the closed-loop context. Such gatekeeping could lead to potentially unwarranted restrictions on access to closed-loop systems, for example, if clinicians are unduly pessimistic regarding future closed-loop outcomes for users seen as “unmotivated.”^11^

Strengths of our study include in-depth interviews with clinicians from different backgrounds serving a range of populations, and with varied experience of closed-loop technology. Our study is limited by uneven numbers of interviewees serving pregnant (*n* = 17), pediatric (*n* = 12), and adult (*n* = 7) populations, and by the dominance of physicians (*n* = 15) as opposed to professions such as nursing (*n* = 8) and dietetics (*n* = 6). Self-selection bias is possible insofar as clinicians who agreed to participate may have been positively disposed toward closed-loop technology and/or participation in research projects. The geographical spread of our interviewees was limited by the low number of clinicians recruited at Hospital 3 (*n* = 3), and because a second clinic at Hospital 3, serving the pediatric population, declined to participate in the study. While we aimed to recruit the widest possible range of participants, it is possible that clinicians working in other contexts may have different views regarding the introduction of closed-loop technologies into mainstream care, especially in hospitals without diabetes technology trial experience and/or nonteaching hospitals. Future research could investigate the views of clinicians working in a wider variety of geographical settings (including settings beyond the United Kingdom), and serving a wider range of populations, including those whose cultural beliefs may present further barriers to closed-loop adoption.^15^ Finally, our study is also limited by the rapid pace of development in the field of diabetes technology, which could lead to different findings if our study was to be repeated in the future, at a time when, for example, people with type 1 diabetes may make widespread use of home-made closed-loop systems.^21^ Our interviewees' concerns regarding calibration, for instance, may be less relevant to future systems.

In conclusion, clinicians expressed a range of opinions regarding eligibility for future closed-loop technology in England. Some emphasized continuity with preceding technologies such as insulin pumps, while others expected either tighter or more liberal access arrangements for closed-loop systems. Since clinicians mediate user access to technology, these varied attitudes may exert substantial impact on future technology use, especially if clinicians are excessively optimistic or pessimistic regarding likely outcomes of closed-loop usage. To optimize technology adoption and equitable uptake, future implementation pathways should consider clinician attitudes toward technology use and access, in addition to the need for tailored education programs to build clinician knowledge of new diabetes technologies. In this context, one possible approach could be to undertake formative consultation with clinicians (alongside other stakeholders) to develop a settled and widely shared technology identity for closed-loop systems, and co-design of closed-loop technology pathways.^22^

## Supplementary Material

Supplemental data

Supplemental data

## References

[1] Boughton CK, Hovorka R: Is an artificial pancreas (closed-loop) system for type 1 diabetes effective? Diabetic Med 2019;36:279–286 30183096 10.1111/dme.13816

[2] Bekiari E, Kitsios K, Thabit H, et al.: Artificial pancreas treatment for outpatients with type 1 diabetes: systematic review and meta-analysis. BMJ 2018;361:k1310 29669716 10.1136/bmj.k1310 PMC5902803

[3] Brown SA, Kovatchev BP, Raghinaru D, et al.: Six-month randomized, multicenter trial of closed-loop control in type 1 diabetes. N Engl J Med 2019;318:1707–1717 10.1056/NEJMoa1907863 PMC7076915 31618560

[4] Messer LH, Berget C, Forlenza GP: A clinical guide to advanced diabetes devices and closed-loop systems using the CARES paradigm. Diabetes Technol Ther 2019;21:462–469 31140878 10.1089/dia.2019.0105 PMC6653788

[5] Callahan D: Taming the Beloved Beast: How Medical Technology Costs are Destroying Our System. Princeton, NJ: Princeton University Press, 2009

[6] Lal R, Basina M, Maahs DM, et al.: 80-OR: 670G clinical experience. Diabetes Care 2019;42:2190–2196 31548247 10.2337/dc19-0855 PMC6868462

[7] Farrington C: Psychosocial impacts of hybrid closed-loop systems: a review. Diabetic Med 2018;35:436–449 29247547 10.1111/dme.13567

[8] NICE (National Institute for Health and Care Excellence): 2019 Surveillance of Diabetes. https://www.nice.org.uk/guidance/ng28/resources/2019-surveillance-of-diabetes-nice-guidelines-ng17-ng18-ng19-and-ng28–6837997933/chapter/Surveillance-decision?tab=evidence (accessed 1212, 2019) 31891469

[9] Greenhalgh T, Wherton J, Papoutsi C, et al.: Beyond adoption: a new framework for theorizing and evaluating nonadoption, abandonment, and challenges to the scale-up, spread, and sustainability of health and care technologies. J Med Internet Res 2017;19:e367 29092808 10.2196/jmir.8775 PMC5688245

[10] Dixon-Woods M, Cavers D, Agarwal S, et al.: Conducting a critical interpretive synthesis of the literature on access to healthcare by vulnerable groups. BMC Med Res Methodol 2006;6:35 16872487 10.1186/1471-2288-6-35 PMC1559637

[11] Farrington C: Clinician attitudes to diabetes technology. Lancet Diabetes Endocrinol 2017;6:15 10.1016/S2213-8587(17)30411-4 29273166

[12] Lawton J, Kirkham J, Rankin D, et al.: Who gains clinical benefit from using insulin pump therapy? A qualitative study of the perceptions and views of health professionals involved in the relative effectiveness of pumps over MDI and structured education (REPOSE) trial. Diabetic Med 2015;33:243–251 26248590 10.1111/dme.12879

[13] Tanenbaum ML, Adams RB, Hanes SJ, et al.: Optimal use of diabetes devices: clinician perspectives on barriers and adherence to device use. J Diabetes Sci Technol 2017;11:484–492 28745093 10.1177/1932296816688010 PMC5505431

[14] Stuckey HL, Vallis M, Burns KK, et al.: ‘I do my best to listen to patients': qualitative insights into DAWN2 (diabetes psychosocial care from the perspective of health care professionals in the second diabetes attitudes, wishes and needs study). Clin Ther 2015;37:1986–1998 26169765 10.1016/j.clinthera.2015.06.010

[15] Lee YK, Lee YP, Ng CJ: A qualitative study on healthcare professionals' perceived barriers to insulin initiation in a multi-ethnic population. BMC Family Pract 2012;13:28 10.1186/1471-2296-13-28 PMC3389339 22469132

[16] Lawton J, Kirkham J, Rankin D, et al.: Who gains clinical benefit from using insulin pump therapy? A qualitative study of the perceptions and views of health professionals involved in the relative effectiveness of pumps over MDI and structured education (REPOSE) trial. Diabetic Med 2015;33:243–251 26248590 10.1111/dme.12879

[17] Lawton J, White D, Rankin D: Staff experiences of closing out a clinical trial involving withdrawal of treatment: qualitative study. Trials 2017;18:61 28173843 10.1186/s13063-017-1813-y PMC5297163

[18] Braun V, Clark V: Using thematic analysis in psychology. Qual Res Psychol 2006;3:77–101

[19] Farrington C, Allen J, Tauschmann M, et al.: Factors affecting recruitment of participants for studies of diabetes technology in newly-diagnosed youth with type 1 diabetes: a qualitative focus group study with parents and children. Diabetes Technol Ther 2016;18:568–573 27355100 10.1089/dia.2016.0157 PMC5035376

[20] Ulucanlar S, Faulkner A, Perice S, Elwyn G: Technology identity: the role of sociotechnical representations in the adoption of medical devices. Soc Sci Med 2013;98:95–105 24331887 10.1016/j.socscimed.2013.09.008

[21] Farrington C: Hacking diabetes. Lancet Diabetes Endocrinol 2017;5:332 27913173 10.1016/S2213-8587(16)30397-7

[22] Farrington C: Co-designing healthcare systems: between transformation and tokenism. J R Soc Med 2016;109:368–371 27729594 10.1177/0141076816658789 PMC5066532

