# Supplementary material for: Who Should Access Closed-Loop Technology? A Qualitative Study of Clinician Attitudes in England
Source: Diabetes Technol Ther. 2020 Apr 27;22(5):404–10. doi: 10.1089/dia.2019.0380 (PMC7196368; doi:10.1089/dia.2019.0380)
Supplement: Supplemental data [file Supp_Datat.pdf]

# Supplementary Data

## Interview Topic Guide

[To be read to interviewees:] The interview has two main parts, focusing on:

- Your attitudes to, and experience of, diabetes technology as used by patients; and
- The organizational and professional culture at your clinic

STRUCTURE:
**Section 1: Diabetes Technology**

(a) Attitudes to technology in general
(b) Attitudes to non-closed-loop technologies
(c) Attitudes to closed-loop technologies

**Section 2: Clinic Culture**

(a) Organizational culture
(b) Multidisciplinary working and team climate
(c) Resources

QUESTIONS:
**Section 1: Diabetes Technology**
(a) Attitudes to technology in general

(1) How would you describe your attitude toward new technology in general, i.e., outside work? (Prompt: would you describe yourself as an early adopter?)
(2) Have you ever used wearable technologies such as activity trackers?
(3) Do you think technology in general makes work easier or more difficult?

Moving on to your clinical experience of diabetes technologies like insulin pumps and CGM:
(b) Attitudes to non-closed-loop technologies

(1) Roughly what proportion of your patients currently use insulin pumps and CGM sensors?
  (a) In what ways have you been involved with these patients in terms of prescribing technology, training processes, advising, and/or troubleshooting?
  (b) If so, what kind of training have you received for this work?
  (c) How did you find the process of learning your way around these technologies?
(2) Are requests for new technologies usually patient led, clinician led, or a mix?
  (a) When patient led: do particular kinds of patients tend to ask for pumps? If so, do they tend to give similar reasons, or do they vary between patients?
  (b) When clinician led: what prompts you or your colleagues to recommend technologies to patients? What role does NICE guidance play in such recommendations?
  (c) Either way: are there sometimes differences between clinician and patient views regarding candidacy? If so, how are these resolved?
(3) What is the process—and how formalized is it—for considering a patient's candidacy for (e.g.) a pump?
  (a) Is there a dedicated pathway for access to pumps/CGM? If so, could you describe it?
  (b) Are there specific meetings to refer patients to pathways?
  (c) What information is brought to bear in candidacy discussions—past experience, specialist knowledge, biomedical data, patient records, personality, and funding situation?
  (d) Do patients ever drop out in the middle of the process? If so, why?
  (e) Are you or your colleagues concerned about the risk of a two-tier population arising, with a divide between those who can access technology and those who cannot?
  (f) What role does NICE guidance play in the process?
  (g) Do you think NICE guidance is open to interpretation? How would you define, for example, ''disabling hypoglycemia''?
(4) Do you think it is possible to predict accurately which patients will benefit from diabetes technologies?
  (a) If so, what kinds of information are most relevant?
(5) When patients start using technologies that are new to them:
  (a) What kind of expectation management is necessary?
  (b) Do you think patients are able to change the way they interact with technology?

(c) Attitudes to closed-loop technologies

(1) Have you ever been involved in closed-loop trials?
  (a) If yes, which studies? How did this experience affect your understanding of, and attitude towards, closed-loop systems?
  (b) If not, can you tell me what you know about closed-loop systems, and how you came across your knowledge (e.g., research article, blogs, and colleagues)?(2) Do you think you would see a similar range of success for patients using closed-loop systems as you would for (e.g.) insulin pumps?
  (a) How might patient experience differ as a result of closed-loop technology, as opposed to insulin pumps and CGM?)
(3) Do you think different discussions would take place in the clinic regarding which patients to recommend for use of closed-loop systems, as opposed to existing technologies?
  (a) If so: which new factors in particular will need to be taken into account?
(4) How confident would you be in terms of advising patients how to use these systems?
  (a) What kind of additional training might be required?

   (b) Would you be more concerned about patients using these systems unsupervised than you are about existing technologies?
   (c) Are there any particular challenges that might emerge with the specific population your clinic serves, or in your geographical area?
(5) Do you think these systems would require the provision of additional support systems, compared to existing technologies?
   (a) If so, what kinds of support in particular?
   (b) Would your clinic be able to provide this support?

**Section 2: Clinic Culture**

(a) Organizational culture

(1) How would you characterize the workplace culture in this clinic?
   (a) Would you describe it as an extended family with lots of personal sharing, or as a place where the main focus is getting the job done?
   (b) Does the clinic have lots of rules and guidelines, or is it more like a dynamic company with room for innovation?

(b) Multidisciplinary working and team climate

(1) Can you describe the management system at the clinic?
(2) Are there any challenges in terms of professions being managed by other professions?
(3) Is there a clear vision shared by the team, or different visions for different professions? How does this affect teamwork?
(4) Can you describe how information sharing works in the clinic?

(c). Resources

(1) Do you feel the clinic has sufficient resources to carry out its work, in terms of (e.g.) staffing, training, and funding for treatments?
(2) Are there particular challenges in your geographical area?
(3) Are there differences in resources for different professions within the clinic?
(4) Do the clinic systems (e.g., IT) support the work of the clinic or are there challenges in this regard?
