# Supplementary material for: Who Should Access Closed-Loop Technology? A Qualitative Study of Clinician Attitudes in England
Source: Diabetes Technol Ther. 2020 Apr 27;22(5):404–10. doi: 10.1089/dia.2019.0380 (PMC7196368; doi:10.1089/dia.2019.0380)
Supplement: Supplemental data [file Supp_Table_S1.pdf]

Supplementary Table S1. Thematic Analysis Coding Framework

Name of code	*No. of participants*	*No. of references*
Access pathway		
Attrition	21	26
Equity	29	39
Format	31	107
Funding	24	51
NICE guidance	26	58
Definitions of terms	10	13
Flexibility	22	30
Gaming	9	15
Precision	14	25
Self-funding	21	25
Stakeholders	10	36
Clinician characteristics		
Profession	36	58
Levels of type 1 contact	22	23
Tech contact	33	62
Tech opinions	33	41
Clinics		
Clinic population	20	45
Information sharing	15	16
Information technology	32	38
Levels of tech usage	34	62
Local challenges	5	7
Management and vision	22	26
Organization	22	30
Organizational culture	33	49
Professional collaboration	26	43
Resources	31	47
Technology culture	26	65
Closed loop		
Changes needed for future care	28	38
Closed-loop candidacy	30	41
Do-it-yourself closed loop	2	4
Envisaged benefit	20	27
Experience of trials	10	11
Knowledge of closed loop	18	19
Patient limitations	22	34
Views on patients		
Adaptability	26	40
Brand differences	17	19
Candidacy	32	165
Expectations	17	24
Predictability	35	63
Proactivity	32	80

NICE, National Institute for Health and Care Excellence.
